# Supplementary material for: PWAS: proteome-wide association study—linking genes and phenotypes by functional variation in proteins
Source: Genome Biol. 2020 Jul 27;21:173. doi: 10.1186/s13059-020-02089-x (PMC7386203; doi:10.1186/s13059-020-02089-x)
Supplement: Supplementary file 9 — Additional file 9: Review history. [file 13059_2020_2089_MOESM9_ESM.docx]

**Review History**

**First round of review**

**Reviewer 1**

Brandes et al propose a gene-based, protein-centered, association method using predicted protein function to prioritize causal genes. Similarly to PrediXcan/TWAS methods, PWAS predicts the function of proteins based on individuals' genetic profile are correlates it with traits, providing mechanistic explanation of the association. They apply the method to 49 UKB phenotypes and find that 23% of their significant associations were not discovered by GWAS alone. Vignettes of genes associated with prostate cancer are shown where the results are consistent with prior biological knowledge.

The paper is overall well written and figures are clear and informative. The method will be a useful addition to the statistical geneticist toolkit. However, the software needs to be made available more readily before the paper gets accepted; available upon request is not acceptable.

Since GWAS loci are mostly located outside of coding regions, it seems that the applicability of this method is restricted. Please provide an estimate of the proportion of GWAS loci that can be attributed to alteration of protein function.

Major issues
--------------
As mentioned above "source code available upon request" is not acceptable

How does the noise parameter epsilon in the protein prediction work? More detail on the meaning of this is needed. A brief description of the prediction model should be included to help the reader understand the meaning of the simulation parameters.

In the colorectal cancer vignette, the switch to q-values seems a bit strange. The associations are not significant at Bonferroni corrected level but given prior knowledge about some of the genes, I don't see a problem highlighting them. But since colorectal cancer trait was selected to describe in more detail, the qqplot should also be shown, even if the significance is rather modest.

Reproducibility of this analysis is unclear to me. Is the FIRM software publicly available? I would like the authors address the reproducibility of the results and the availability of the software necessary to conduct the study described in this manuscript.

The number of schizophrenia genes (114 collectively and 80 for the GWAS) seems unrealistic given the extremely small number of schizophrenia cases. Is this because you are only considering coding variants?

The qq plot of some of the continuous phenotypes seem to be almost vertical suggesting some kind of artefact. What is the interpretation? Are all genes significant for these?

Can you explain what you mean by "the reliance of PWAS on rigorous statistics keeps in insensitive to false discoveries even with imperfect genotyping"?

Protein function prediction is also noisy, it is not clear to me why you claim that "PWAS assigns scores in a deterministic, consistent manner". Please clarify.

"making the selection of a relevantreference panel for expression-based methods a daunting task" also seems unsupported. Availability of expression in multiple tissues is beneficial rather than detrimental. One can aggregate the evidence across contexts and tissues. Please clarify.


Minor issues
-------------
It would be good to add a discussion of the limitations of PWAS given that the majority of GWAS hits are located in non-coding regions. Perhaps a combined analysis of coding and non-coding regions could be done using both predicted expression levels and function.

CDS used in Figure 2 but not defined.

**Reviewer 2**

===
The authors have proposed PWAS (Proteome-wide association study) to detect coding genes that are associated with the trait of interest. I think the paper has interesting ideas and the biological finding is interesting. However, I do have some concerns:

1. The work is very interesting, but the idea of PWAS is highly similar to TWAS (transcript-wide association study). The authors have used proteome instead of gene expression which make the work lack novel method. In real and simulated datasets the authors need to compare their results with TWAS. It is important for the reader to know which of these genes are not detected by TWAS and FUSION (Mancuso et al. Nature Genetics 2018). FUSION can detect the causal genes using TWAS results.

2. Most of GWAS variants fall in non-coding region of the genome. I am not sure how the authors utilize any non-coding variants. So it is interesting that the GWAS did not detect additional genes compared to PWAS.

3. This work is submitted to ISMB as I am reviewer both papers. I am not sure why the authors have done. ISMB unlike RECOMB prohibit parallel submission the work to another journal. I think the authors have mixed up RECOMB and ISMB rules.

4. The authors did not provide their code so the reviewers can check the code.

Minor comments:

1. The proposed method require individual level genotype and phenotype data. Please make it clear in the paper.
2. The authors need to cite the following work which explain PWAS:
a. Okada et al. Nature communication 2016. doi: 10.1038/ncomms12649
b. Butter et al. Plos Genetics 2012. doi:10.1371/journal.pgen.1002982

**Authors’ response to reviewers**

Reviewer #1

Brandes et al propose a gene-based, protein-centered, association method using predicted protein

function to prioritize causal genes. Similarly to PrediXcan/TWAS methods, PWAS predicts the function of

proteins based on individuals' genetic profile are correlates it with traits, providing mechanistic

explanation of the association. They apply the method to 49 UKB phenotypes and find that 23% of their

significant associations were not discovered by GWAS alone. Vignettes of genes associated with prostate

cancer are shown where the results are consistent with prior biological knowledge.

We thank the reviewer for the highly relevant comments, which helped improving the quality of our

work and clarify our writing.

The paper is overall well written and figures are clear and informative. The method will be a useful

addition to the statistical geneticist toolkit. However, the software needs to be made available more

readily before the paper gets accepted; available upon request is not acceptable.

We fully agree. For the past month we have put concentrated effort on organizing and documenting our

code, now having PWAS finally available as an open source project with simple command line interface

(https://github.com/nadavbra/pwas). We also open sourced our data processing pipeline

(https://github.com/nadavbra/ukbb_parser).

We now link to the GitHub repositories in the paper. We also included a full list of the open source

projects that are part of this work at the end of the Supplementary Methods.

To make sure that the results reported in this study are fully consistent with the version of PWAS

available on GitHub, we re-analyzed all the associations with the latest version of PWAS, which resulted

minor changes in the summary statistics reported across the tables and figures. The broad picture of our

work and its conclusions remain unchanged.

Since GWAS loci are mostly located outside of coding regions, it seems that the applicability of this

method is restricted. Please provide an estimate of the proportion of GWAS loci that can be attributed

to alteration of protein function.

This is correct. PWAS deals only with coding genes. To make sure that the reader doesn’t overlook that,

we added the following sentence to the discussion:

“It should also be kept in mind that most GWAS associations occur in non-coding regions [49], which

PWAS completely ignores in its current form.”

Major issues

--------------

As mentioned above "source code available upon request" is not acceptable

See our response above.

How does the noise parameter epsilon in the protein prediction work? More detail on the meaning of

this is needed. A brief description of the prediction model should be included to help the reader

understand the meaning of the simulation parameters.

Indeed, we didn’t properly explain the meaning of the noise parameter in the simulation analysis. To

better clarify it to the reader, we included the following sentence in the Results section:

“Specifically, we distorted the variant effect scores predicted by FIRM (in the range between 0 to 1) with

additive Gaussian noise of standard deviation 𝜖.“

Full mathematical explanation of the simulations is provided in the "Simulation analysis" section in the

Supplementary Methods.

In the colorectal cancer vignette, the switch to q-values seems a bit strange. The associations are not

significant at Bonferroni corrected level but given prior knowledge about some of the genes, I don't see

a problem highlighting them. But since colorectal cancer trait was selected to describe in more detail,

the qqplot should also be shown, even if the significance is rather modest.

Letting GWAS use Bonferroni correction and PWAS use FDR is the proper way to compare the two

methods (given these are the significance thresholds used for each of the two). To make sure that

readers notice this subtle point you have eluded to, we added the following sentence:

“Notably, while GWAS determines significance by the Bonferroni-corrected significance level (p < 5E-07

for coding regions), PWAS determines significance by FDR (q < 0.05), another clear advantage of PWAS

as a gene-based test.”

The full QQ plot of PWAS for colorectal cancer (over all ~18K tested genes) is provided in Fig. 4b. The

GWAS QQ plot appears in Supplementary Fig. S7.

Reproducibility of this analysis is unclear to me. Is the FIRM software publicly available? I would like the

authors address the reproducibility of the results and the availability of the software necessary to

conduct the study described in this manuscript.

We added to the Methods a link to the FIRM software, which is publicly available. As mentioned above,

we made sure to provide full details to all used software, which are now open sourced and well

documented.

The number of schizophrenia genes (114 collectively and 80 for the GWAS) seems unrealistic given the

extremely small number of schizophrenia cases. Is this because you are only considering coding

variants?

We tested this possibility. A small number of cases doesn’t necessarily rule out significant results. For

example, with lupus we have even a lower number of cases (only 264), yet we obtain 164 significant

genes (mostly through standard GWAS as well). These significant variants show strong overlap with

known associations (e.g. from the GWAS Catalog, see Supplementary Table S7), so we feel quite

confident that our methods are solid. In the case of schizophrenia, it is indeed possible that most of the

signal cannot be attributed to coding part of the genome, and, therefore, it is possible that most of the

significant genes we discover are the result of linkage disequilibrium.

The qq plot of some of the continuous phenotypes seem to be almost vertical suggesting some kind of

artefact. What is the interpretation? Are all genes significant for these?

When highly polygenic traits are involved (e.g. height, BMI, blood counts), it is expected to have a very

strong signal leading to vertical-looking QQ plots (especially in cohorts as large as the UK Biobank). This

is not unique to our method, and a similar pattern emerges with standard GWAS (Supplementary Fig.

S7). A similar pattern can also be seen in the works of others (e.g. see https://doi.org/10.1038/s41588-

018-0248-z via http://geneatlas.roslin.ed.ac.uk/). Note that in much of the existing literature, QQ plots

don’t look “vertical” due to distortion of the scale (using a different scale for the x and y axes on the

plots). We think that this distorted way of visualization is misleading (failing to capture the polygenic

signal of phenotypes), and therefore chose to show all our QQ plots at the right scale.

Can you explain what you mean by "the reliance of PWAS on rigorous statistics keeps in insensitive to

false discoveries even with imperfect genotyping"?

We revised the sentence to make it clear that we do not claim any advantage over existing methods (in

this respect):

“It should be noted that while more accurate genotyping should indeed enhance its statistical power,

the reliance of PWAS on rigorous statistics keeps it robust to false discoveries as other GWAS methods,

even with imperfect genotyping.”

Protein function prediction is also noisy, it is not clear to me why you claim that "PWAS assigns scores in

a deterministic, consistent manner". Please clarify.

See our next point.

"making the selection of a relevant reference panel for expression-based methods a daunting task" also

seems unsupported. Availability of expression in multiple tissues is beneficial rather than detrimental.

One can aggregate the evidence across contexts and tissues. Please clarify.

We revised the entire paragraph, which now reads:

“Contrary to expression-based methods, PWAS assigns gene scores that depend only on genotypes.

Gene expression is highly volatile, with substantial variability between tissues, epigenetic conditions and

many other non-genetic factors. TWAS, for example, requires the researcher to choose a target tissue

for the association tests (e.g. based on prior assumptions about the phenotype under study). Damage to

protein products, on the other hand, is mostly a direct result of genetic makeup. This benefits PWAS in

two major ways. First, it offers reduced computational complexity, since it is sufficient to compute the

gene score matrices only once, which can later be used for any phenotype. More importantly, it relieves

the researcher from the need to select a specific tissue or expression profile for the analysis. Indeed,

most human traits are not confined to specific tissues, let alone specific cellular conditions, making the

selection of a relevant reference panel for expression-based methods non trivial.”

We hope the new phrasing is clearer.

Using an expression-based method (e.g. TWAS) for a phenotype not confined to a specific tissue is

indeed a non-trivial task. On the computational side, running on a large set of contexts and tissues

would require more pre-processing and subsequent statistical tests (to predict each of the expression

profiles, and then test for their associations with the phenotype). From a statistical perspective, it would

probably also require some sort of multiple testing correction.

Minor issues

-------------

It would be good to add a discussion of the limitations of PWAS given that the majority of GWAS hits are

located in non-coding regions. Perhaps a combined analysis of coding and non-coding regions could be

done using both predicted expression levels and function.

We added the following sentence to the discussion:

“It should also be kept in mind that most GWAS associations occur in non-coding regions [49], which

PWAS completely ignores in its current form.”

When discussing PrediXcan and TWAS, we also write:

“We consider PWAS complementary to methods that model other functional aspects of the genome.”

We fully agree that a combined analysis of coding and non-coding regions could be done using both

predicted expression levels and function. This is indeed something we hope to see in the near future.

We encourage our readers to engage with this sort of analysis, and we also keep it as a future work for

ourselves.

As the paper is very focused on methodology and comparative benchmarking (i.e. comparing PWAS

head-to-head with GWAS and SKAT), we think that introducing such an analysis here would not do it

justice, given the scope of this work.

CDS used in Figure 2 but not defined.

We added an explanation in the figure legend.

Reviewer #2

The authors have proposed PWAS (Proteome-wide association study) to detect coding genes that are

associated with the trait of interest. I think the paper has interesting ideas and the biological finding is

interesting. However, I do have some concerns:

1. The work is very interesting, but the idea of PWAS is highly similar to TWAS (transcript-wide

association study). The authors have used proteome instead of gene expression which make the work

lack novel method. In real and simulated datasets the authors need to compare their results with TWAS.

It is important for the reader to know which of these genes are not detected by TWAS and FUSION

(Mancuso et al. Nature Genetics 2018). FUSION can detect the causal genes using TWAS results.

While TWAS works on expression data, PWAS considers genetic damage to the function of the protein

products themselves (e.g. through changes to the structures of the proteins and their binding potential).

This is a completely distinct molecular property, fully orthogonal to expression (either at the transcript

or protein level). Therefore, while a comprehensive analysis considering the two methods as

complementary might be interesting as future work (we elude to this possibility in the discussion and

encourage it), we think there is no room for direct comparison between the two approaches, as they do

not attempt to capture the same biological signal. We have no doubt that TWAS is capable of finding

many associations that PWAS is completely blind to.

We made extra efforts to avoid confusion between PWAS and TWAS throughout the manuscript. We

also added a reference to Mancuso et al. Nature Genetics 2018.

2. Most of GWAS variants fall in non-coding region of the genome. I am not sure how the authors utilize

any non-coding variants. So it is interesting that the GWAS did not detect additional genes compared to

PWAS.

Our entire analysis throughout this paper focuses solely on protein-coding genes, also when using

standard GWAS. PWAS indeed doesn’t utilize non-coding variants and completely ignores them. You are

right to point out that GWAS is capable of finding many non-coding associations (and also non-coding

genes) that PWAS is completely unable to deal with, but these are not part of our analysis.

To make the scope of our work clearer to the reader, we added the following sentence in the discussion:

“It should also be kept in mind that most GWAS associations occur in non-coding regions [49], which

PWAS completely ignores in its current form.”

3. This work is submitted to ISMB as I am reviewer both papers. I am not sure why the authors have

done. ISMB unlike RECOMB prohibit parallel submission the work to another journal. I think the authors

have mixed up RECOMB and ISMB rules.

There was indeed a misunderstanding with ISMB. Our work was sent to the wrong track. Following

ISMB’s advice, we moved it to the late-breaking track. Anyway, this issue has already been clarified with

both ISMB’s committee and the editors of Genome Biology.

4. The authors did not provide their code so the reviewers can check the code.

This was indeed inappropriate, and we fixed it. For the past month we have put concentrated effort on

organizing and documenting our code, now having PWAS finally available as an open source project with

simple command line interface (https://github.com/nadavbra/pwas). We also open sourced our data

processing pipeline (https://github.com/nadavbra/ukbb_parser).

We now link to the GitHub repositories in the paper. We also included a full list of the open source

projects that are part of this work at the end of the Supplementary Methods.

To make sure that the results reported in this study are fully consistent with the version of PWAS

available on GitHub, we re-analyzed all the associations with the latest version of PWAS, which resulted

minor changes in the summary statistics reported across the tables and figures. The broad picture of our

work and its conclusions remain unchanged.

Minor comments:

1. The proposed method require individual level genotype and phenotype data. Please make it clear in

the paper.

We edited the Discussion to make it clearer. See the entire paragraph beginning with the sentence:

“A potential limitation of PWAS is its reliance on complete individual-level data (including genotype and

phenotype information).”

2. The authors need to cite the following work which explain PWAS:

a. Okada et al. Nature communication 2016. doi: 10.1038/ncomms12649

b. Butter et al. Plos Genetics 2012. doi:10.1371/journal.pgen.1002982

It is unfortunate that the PWAS acronym has been used in other contexts.

The first reference (Okada et al.) describes a method dealing with protein expression (unlike our method

that deals with protein function, and ignores expression at either the transcript or protein level). The

second reference (Butter et al.) also describes a completely unrelated method, defining PWAS as

“proteome-wide analysis of SNPs”, which is “a technique to study differential transcription factor

binding to nucleotide sequences”.

We think that mentioning these unrelated works will only lead to confusion for our readers

**Second round of review**

**Reviewer 1**

My main concern was addressed, which was the availability of the software.

A few comments regarding the response from the authors

1) "PWAS determines significance by FDR (q < 0.05), another clear advantage of PWAS as a gene-based test.”
 Using FDR is not a benefit of PWAS, but a decision you or the user makes. Please remove this sentence which is misleading.

2) "A small number of cases doesn’t necessarily rule out significant results "

Given what we know about the genetic architecture of SCZ, a small number of cases does preclude significant discoveries. Something doesn't seem right. Lupus is an autoimmune disorder, known to have very large effect loci, which can be discovered with relatively small number of cases.

**Authors’ response to reviewer**

1) "PWAS determines significance by FDR (q < 0.05), another clear advantage of

PWAS as a gene-based test.”

Using FDR is not a benefit of PWAS, but a decision you or the user makes. Please

remove this sentence which is misleading.

Response: We removed this part of the sentence.

2) "A small number of cases doesn’t necessarily rule out significant results "

Given what we know about the genetic architecture of SCZ, a small number of cases

does preclude significant discoveries. Something doesn't seem right. Lupus is an

autoimmune disorder, known to have very large effect loci, which can be discovered

with relatively small number of cases.

Response: We checked it again, and the results look reliable.
